# Unravel the Local Complexity of Biological Environments by MALDI Mass Spectrometry Imaging

**DOI:** 10.3390/ijms222212393

**Published:** 2021-11-17

**Authors:** Elvira Sgobba, Yohann Daguerre, Marco Giampà

**Affiliations:** 1 Department of Forest Genetics and Plant Physiology, Swedish University of Agricultural Sciences, 90183 Umeå, Sweden; elvira.sgobba@slu.se (E.S.); yohann.daguerre@slu.se (Y.D.); 2 Department of Clinical and Molecular Medicine, Norwegian University of Science and Technology, 7491 Trondheim, Norway

**Keywords:** MALDI MSI, metabolomics, imaging, plants, microbiology, lipidomics

## Abstract

Classic metabolomic methods have proven to be very useful to study functional biology and variation in the chemical composition of different tissues. However, they do not provide any information in terms of spatial localization within fine structures. Matrix-assisted laser desorption ionization mass spectrometry imaging (MALDI MSI) does and reaches at best a spatial resolution of 0.25 μm depending on the laser setup, making it a very powerful tool to analyze the local complexity of biological samples at the cellular level. Here, we intend to give an overview of the diversity of the molecules and localizations analyzed using this method as well as to update on the latest adaptations made to circumvent the complexity of samples. MALDI MSI has been widely used in medical sciences and is now developing in research areas as diverse as entomology, microbiology, plant biology, and plant–microbe interactions, the rhizobia symbiosis being the most exhaustively described so far. Those are the fields of interest on which we will focus to demonstrate MALDI MSI strengths in characterizing the spatial distributions of metabolites, lipids, and peptides in relation to biological questions.

## 1. Introduction

The understanding of the chemistry of biological structures or compartments is of great interest for biochemists, structural biologists, and pathologists to explain several kinds of biological processes, such as neurotransmission, the mode of action of a drug on a specific biological target, pathogenesis, and microbial interactions. Many approaches such as microscopy, spectroscopy, and several integrating imaging tools are employed to image the biological processes or biological structures, but they do not give an accurate chemical composition of the biological compartment [1,2,3]. Matrix-assisted laser desorption ionization mass spectrometry imaging (MALDI MSI) is an outstanding tool used to extract molecular information by direct desorption of the molecules from the sample surface and to determine their specific mass-to-charge ratio (*m/z*) values. However, besides MALDI MSI, other desorbing and ionization approaches are also worth mentioning, such as desorption electrospray ionization (DESI), secondary ion mass spectrometry (SIMS), and laser ablation electrospray ionization (LAESI), which are employed in MSI procedures accordingly to the desired spatial resolution [4] and are further combined with laser capture microdissection for accurate molecular identification [5].

MALDI MSI, which is a label-free multicomponent imaging analysis, consists of applying a small UV-absorbing compound, so-called matrix, on the sample and on the laser rasterization of every point analyzed with a specific raster width or spatial resolution. In detail, laser irradiation of the analyte–matrix mixtures, present at each point of the analyzed sample, leads to the formation of a highly dynamic and complex expanding plume and the subsequent production of ions by reactions in the gas phase (e.g., proton and/or electron transfer) [6,7]. Then, ions produced by MALDI are detected by a mass spectrometer, e.g., time of flight (TOF) or Fourier transform ionic cyclotronic resonance (FT-ICR). For each point of the sample, mass spectra acquisition generates multiple heat maps. Although the precise mechanism of MALDI processes is still unknown, researchers have proposed several models such as the lucky survivors model [8], the coupled chemical and physical dynamics model [9], or the sublimation driven ionization model [10]. In MALDI MSI, the chemistry of the matrix plays an essential role in the desorption and ionization [11,12], and many improvements have been performed to increase the sensitivity and expand the chemical coverage [13,14,15]. Another parameter to consider in MALDI MSI is the spatial resolution, which depends on the diameter of the laser. Indeed, the spatial resolution defines the structural level of the biological compartments under investigation. Until now, MALDI MSI has been able to reach a spatial resolution of 0.25 μm by using a specific laser setup to localize chemicals at the cellular level [16]. Nevertheless, using a small laser beam can negatively affect ion intensity and molecular coverage [17], leading to the need for highly sensitive detectors [18]. Moreover, the crystal size of the matrix needs to be considered in relation to the diameter of the laser beam when high-spatial-resolution MSI is targeted. The use of new MALDI matrices with amorphous morphology such as polymer matrix [19] and maleic anhydride proton sponge [20] represents a promising strategy to face the crystal size limitations of classical matrices. Finally, the compounds that have been imaged need to be identified by accurate mass and/or molecular fragmentations. This can be achieved by analysis of the desorbed molecules during MALDI MSI acquisition or by analysis of tissue extracts using hyphenated methodologies (e.g., liquid/gas chromatography MS) [21,22].

**Figure 1:**
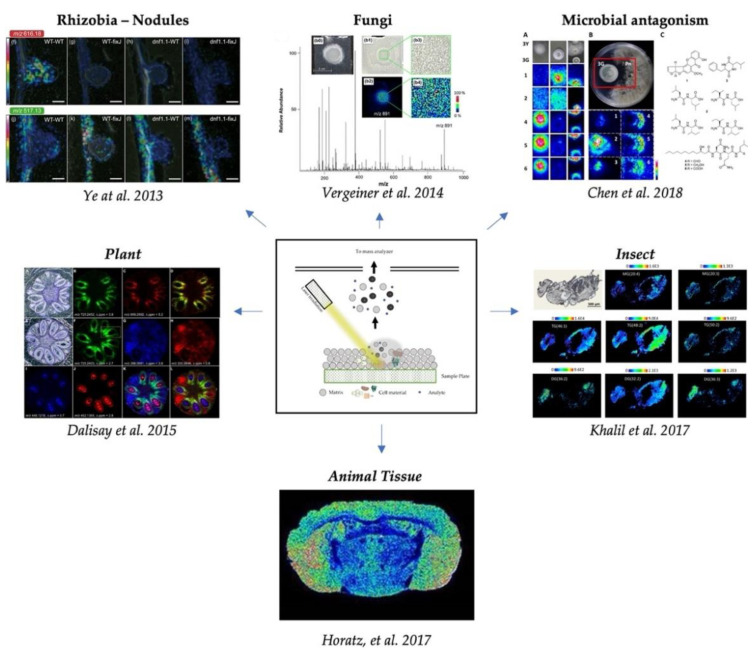
MALDI MSI applied in different areas of research: plant–rhizobia interaction [23], plant development [24], microbial interaction, e.g., antagonism [25], mycology [26], entomology [27], and animal science [28].

In this review, the potential of MALDI MSI to spatially resolve and identify molecules belonging to biological environments will be shown by emphasizing several examples from plant to animal and insect tissue and microbiological interactions (Figure 1). In this review, the corresponding *m/z* values, ionic adducts, and identification method of each compound mentioned are annotated in Supplementary Table S1.

## 2. Metabolites Associated with Microbial Interactions and Antagonism

The processes involved in the antagonistic behavior of microorganisms towards pathogens are diverse and can lead to the following effects: direct parasitism and death of the pathogen, food and space competition with the pathogen, or toxic effects on the pathogen by the release of antibiotic substances by the antagonist [29].

In this context, MALDI MSI is a powerful tool that can be used for the analysis of complex microbial networks. Indeed, a set of secondary metabolites produced by the bacterium *Streptomyces* sp. (CB0028) to control the fungal parasite *Escovopsis* sp. (CBAcro424), both isolated from the nest of the fungus-growing ant *Acromyrmex echinatior*, were identified. MALDI MSI and MS/MS in positive mode allowed to localize and identify different siderophores that have been previously associated with antimicrobial and parasitic mechanisms: desferrioxamine B, ferrioxamine B, and ferrioxamine E [30]. MALDI MSI has also been applied to obtain detailed pictures of the intimate mutualistic interaction between the fungus-growing ants and *Pseudonocardia*, a bacterium that lives on its exoskeletons, and protects its fungal garden from harmful pathogens such as *Escovopsis*. Ergothioneine was detected in MALDI MSI only in the presence of *Pseudonocardia* on the ant propleural plate but its presence did not depend on *Escovopsis* exposure [31,32]. This unique compound might play a role as an antioxidant and is synthesized only by certain species of bacteria belonging to Cyanobacteria, Actinobacteria and Mycobacteria, and certain fungi including Ascomycetes and Basidiomycetes [31].

**Figure 2:**
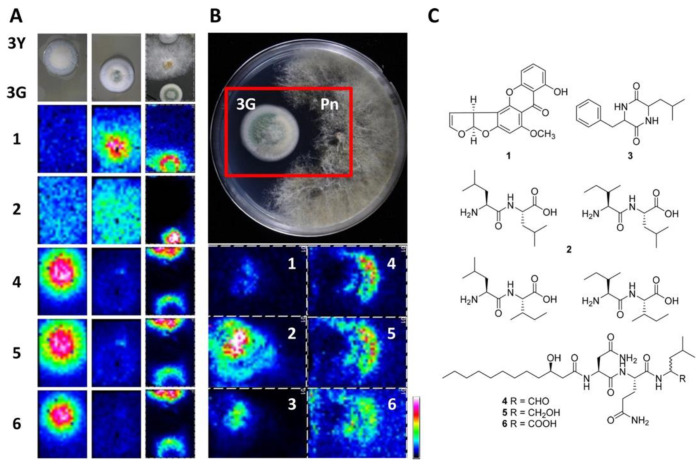
(**A**) Microbial MSI of *Aspergillus* strains (3Y and 3G) cocultured with *Phellinus noxius.* Inhibition zones are shown in the panel (**B**), whereas panel (**C**) depicts structures of *Aspergillus* metabolites produced in response of *P. noxius*: *m/z* 325, [M + H]^+^: sterigmatocystin (1); *m/z* 245, [M + H]^+^: L-Leu-L-Leu, L-Ile-L-Leu, L-Leu-L-Ile, and L-Ile-L-Ile (2); *m/z* 261, [M + H]^+^: cyclo(Phe-Ile) or cyclo(Phe-Leu) (3); *m/z* 578, [M + Na]^+^: fellutamide B (4); *m/z* 580, [M + Na]^+^: fellutamide C (5); *m/z* 594, [M + Na]^+^: new fellutamide (6) [25].

MALDI MSI has been quite successful in the simultaneous visualization of multiple metabolites at the spreading zone of microorganisms. This becomes particularly important in fungi that possess thick and rough colonies, as well as for those requiring complex growing media [33]. Indeed, MALDI MSI in positive mode was successfully applied to identify and predict antifungal metabolites produced during the coculture of two fungi, *Penicillium polonicum* and *Fusarium oxysporum*, at the proximity of the microbial colonies. The two antifungal metabolites identified belonged to the indol alkaloids family and were referred to as fructigenine A and fructigenine B, as shown in Supplementary Table S1 [34]. A similar study applied nano silicone structures to study the interactions between *Phellinus noxius*, a pathogen responsible for brown root rot disease in woody plants [35], and *Aspergillus* strains. Here, it was possible to detect and identify by UPLC-MS/MS a polyketide (e.g., sterigmatocystin) as well as di- and tripeptides (e.g., Fellutamide C) secreted by *Aspergillus* in the inhibitory region around the fungal mycelium [25], as shown in Table 1 and Figure 2.

Siderophores are a class of molecules that represent one among many natural products that bacteria and fungi can secrete to inhibit each other’s growth and development [36]. Pyochelin is a siderophore produced by different *Pseudomonas* and *Burkholderia* species which has been shown to have antifungal properties. It was possible to show via MALDI MSI that the pyochelin secreted by *Burkholderia cenocepacia* 869T2 is esterified by the fungal plant pathogen *Phellinus noxius* 2252 to produce pyochelin-GA. Esterification of siderophores acts as a defense mechanism by reducing the chelation properties of those molecules and ultimately their antifungal effect. Moreover, the concentration of dehydroergosterol peroxide (DHEP) at the edge of the fungal colony was shown to be specifically induced by pyochelin, independently of its esterification status, but this was unrelated to the accumulation of ROS induced by different iron chelators (Table 1) [37].

The chemistry of the matrix applied in MALDI MSI as well as the modality of its application can facilitate the ionization of molecules of certain chemical classes [38]. Indeed, consecutive short spray pulses of the matrix solution formed an evenly closed matrix layer on dried agar, improving the detection of a cytotoxic secondary metabolite pellasoren and a macrocyclic peptide microsclerodermin M produced by *Sorangium cellulosum* So ce38 [39]. This pulsed matrix spray ensured a highly reproducible peak shape and *m/z* value compared to the spectra acquired at high abundant positions all over the colony, as shown in Table 1 [39].

In polymicrobial interactions, microbes can temper with each other’s defense reactions and secrete metabolites reducing their respective progression. Using MALDI TOF and MALDI FT ICR imaging mass spectrometry combined with MS/MS, it was possible to gain new insights into the interaction of the Gram-negative bacteria *Pseudomonas aeruginosa* and the fungus *Aspergillus fumigatus* [40,41]. As a result, the traffic of metabolites and their conversion at the interspace between the two microbes could be visualized. *P. aeruginosa* produced different phenazines (e.g., phenazine-1-carboxamide, PCA), quinolones, and rhamnolipids, known to be involved in quorum sensing and signaling or acting as virulence factors against different microbes, e.g., fungi (Supplementary Table S1) [42]. On the other side, *A. fumigatus* converted the metabolite PCA into 1-hydroxyphenazine (1-HP), 1-methoxyphenazine (1-MP), phenazine-1-sulfate, and phenazine dimers. This was confirmed by the localization of those compounds in the surrounding of *A. fumigatus* rather than *P. aeruginosa*. Although phenazine-1-sulfate had no antifungal activities compared to PCA, the intermediates 1-HP and 1-MP showed increased toxicity, suggesting that the bioconversion of PCA into phenazine-1-sulfate may not be part of a detoxification mechanism [40] (Table 1). Nonetheless, the specific localization of phenazine dimers at the interface between *P. aeruginosa* and *A. fumigatus* suggested that the fungus might temper with the bacterial quorum sensing signaling, iron acquisition and biofilm formation by biotransforming phenazines pyocyanin (PYO) and PCA.

Some bacteria establish parasitic interactions with fungi. They can produce natural products that stimulate morphological changes in fungi such as the formation of chlamydospores harboring bacteria that can act as a plant pathogen vector. Using MALDI MSI, ralsolamycin, a cyclo-lipopeptide produced by *Ralstonia solanacearum*, was shown to stimulate fungal sporulation with thick-cell-wall spores, promote fungal hyphal invasion, and extend fungal survival [43].

## 3. Spatial Localization of Fungal Metabolites

A lot of pathogenic fungi are plant parasites. MALDI MSI represents a powerful tool to investigate biosynthetic pathways on intact sections of infected tissue and get a deeper picture of the parasite–host interaction. *Claviceps purpurea* is a parasite of *Secale cereale* and accumulates highly toxic ergot alkaloids in the sclerotia. MALDI MSI data could provide further insight on the localization function of two alkaloids in the sclerotia: (1) ergocristine (Table 1) localized at the proximal region increasing towards the center where cells undergo morphological changes during the parasitic development, while (2) ergometrine (Table 1) was found evenly distributed within the sclerotia [44].

**Figure 3:**
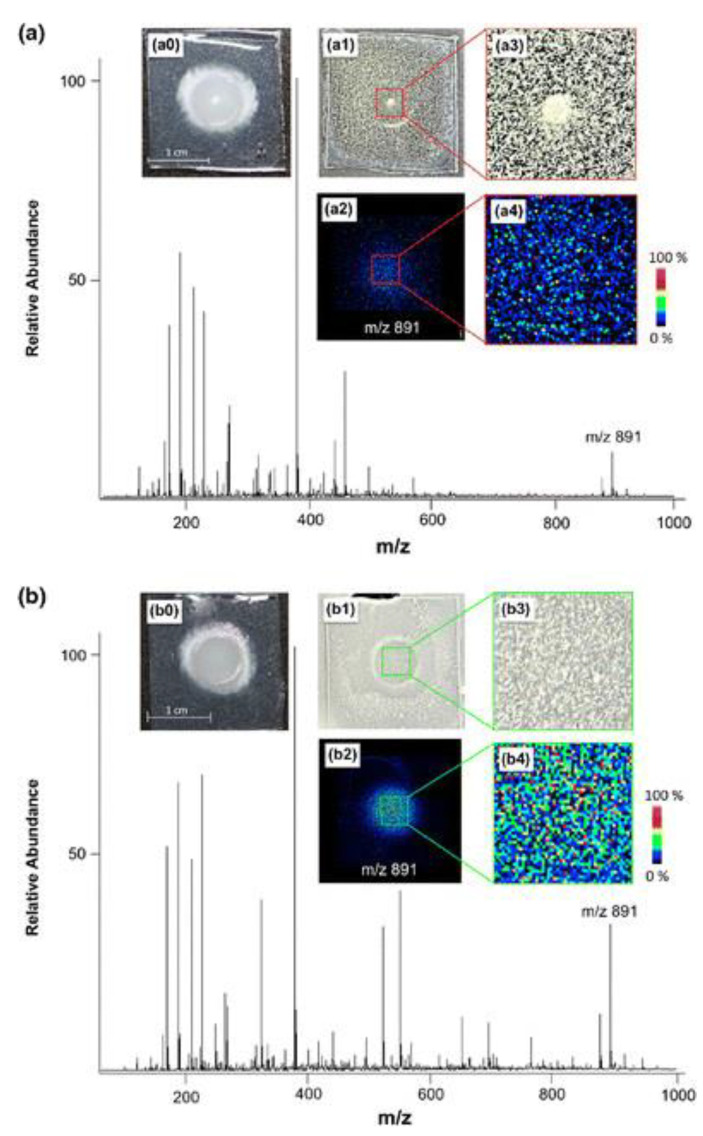
MALDI MSI of *Aspergillus fumigatus* grown on agar (24 h at 37 °C), and influence of matrix application on sensitivity and spatial resolution. (**a**) *A. fumigatus* culture (optical image a0) using dry-coating application of a 1:1 mixture of DHB and CHCA (optical image a1 after matrix application and magnification a3); (**b**) *A. fumigatus* culture (optical image b0) using coating with a dispersed 1:1 mixture of DHB and CHCA in CHCl_3_ (optical image b1 after matrix application and magnification b3). In both panels, the relative distribution (ion images a2 and b2 and their respective magnifications a4 and b4) and the average mass spectrum of dfTafC are depicted (*m/z* 891, [M + K]^+^) [26].

As above mentioned, MALDI matrix deposition development is a growing field to target more complex biological materials. The application of a thin layer of a matrix mixture of 2,5-dihydroxybenzoic acid (DHB) and alpha-Cyano-4-hydroxycinnamic acid (CHCA) on a culture of *A. fumigatus* enabled the detection of the siderophore desferri-triacetylfusarinine (dfTafC) around the colony during iron starvation, as shown in Table 1 and Figure 3 [26].

Another secondary metabolite produced by fungi belongs to the family of solanapyrones [45]. As an example, solanapyrones A, a polyketide-derived secondary metabolite produced by *Ascochyta rabiei*, showed antifungal properties against saprobic competitors and was detected at the inhibitory zone as additional proof of its antifungal activity [46] (Table 1).

Fungi can also produce different natural products beneficial in medicine and agriculture [47]. Indeed, fungi can be used as bio-control agents against different plant pathogens. Via MALDI TOF MSI, it was possible to identify a new antifungal agent lipopetaibol (leucinostatin Z) produced by *Purpureocillium lilacinum* at the inhibitory zone against *Botrytis cinerea*, a pathogen responsible for fruit and vegetable molding [48].

## 4. Spatial Localization of Plant Metabolites

**Figure 4:**
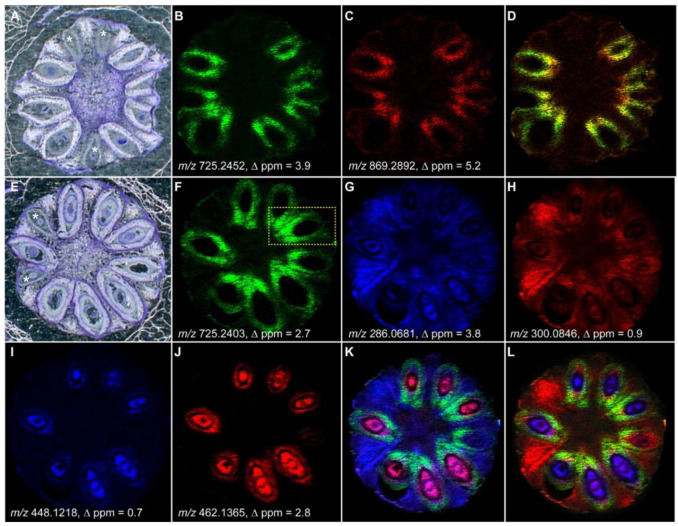
MALDI MSI of the spatial distribution of flax lignans SDG and 6a-HMG SDG 6 days after flowering (DAF) and cyanogenic glucosides linamarin, lotaustralin, linustatin, and neolinustatin 7 DAF. (**A**) Optical image of the transversal section of a flax capsule 6 DAF; (**B**,**C**) MALDI MSI of SDG and 6a-HMG SDG 6 DAF, respectively; (**D**) merge picture of (**B**,**C**); (**E**) an optical image of the transversal section of a flax capsule 7 DAF; (**F**–**J**) MALDI MSI of SDG, linamarin, lotaustralin, linustatin, and neolinustatin, respectively; (**K**) merged picture of (**F**,**G**,**I**); (**L**) merged picture of (**F**,**H**,**J**); Images were generated using a 20 μm spatial resolution, scale: 5 mm [24].

MALDI MSI is a powerful tool for plant biology, constantly evolving to better adapt to the complexity of the samples and the problems of the field. The initial tissue structural limitations of MALDI MSI in plants science have been overcome, and MALDI MSI has been widely applied to different plant tissue and organs, e.g., root, stem, leaf, flower, fruit, and seed, as well as to image a large portfolio of molecules, e.g., peptides, proteins, lipids, flavonoids, and ginsenosides [49], as shown in Supplementary Table S1. As a result, higher-spatial-resolution information during plant growth and development could be accessed. For example, flax lignans secoisolariciresinol diglucoside (SDG) and 6a-hydroxymethylglutaryl secoisolariciresinol diglucoside (6a-HMG SDG) were mostly detected in the seeds’ coat 6–7 days after flowering (DAF) but not at later developmental stages due to their further conversion to higher-molecular-weight phenolics. The plant defense metabolites cyanogenic glucosides, e.g., linamarin and lotaustralin, were detected between 0–7 DAF but not anymore after 10–12 days in all flax capsule tissues, including ovary, seed coat, and embryo tissues. In contrast, linustatin was specifically located in the seed embryo and endosperm at all developmental stages, while neolinustatin was only observed after 7 DAF. Figure 4 illustrates the spatial localization of lignans and cyanogenic glucosides 6–7 DAF. Therefore, MALDI MSI analysis confirmed that the chemical structure and spatial localization of lignans and cyanogenic glucosides were subject to changes during the maturation process of flax capsules (Supplementary Table S1) [24].

Plant alkaloid content has been associated with plant development, fruit maturation, and plant defense mechanisms. In terms of evolution, they are meant to protect but also to ensure that the fruits are not consumed before they are ripe. Some of them are a matter of food safety, while others present an interest as antimicrobial bioactive molecules. In *Lycopersicon esculentum*, for example, aglycon tomatidine showed a very specific pattern in the developing flower, gradually increasing from the locular tissue to the placenta and concentrating in the ovary area and the sepals. Trisaccharide β-tomatine was mostly found inside the cotyledon within the seed. In *Slolanum nigrum*, a drastic decrease of all the alkaloid detected (Solasodine, γ-solasonine, β1-, solasonine, β2-solasonine, α-chaconine, α-solanine, and α-solasonine) was observed in the mesocarp tissue of the fruit during ripening. For comparison, in *Solanum dulcamara*, the same was shown but α- and β-solamarines could still be detected in ripe fruits. Interestingly, although present in high concentrations in the surrounding mesocarp of unripe fruit, α-, β-, and γ-solamarines were absent from the seeds [50]. Finally, in potato tubers, toxic glycoalkaloids, e.g., dehydrosolanine, α-solanine, dehydrochaconine, and α-chaconine, were shown to accumulate upon storage in the sprout and the periderm but less in the medulla. The highest levels were detected in the sprout [51].

Germinated rice is commonly sold as enriched in gamma-aminobutyric acid (GABA) and essential amino acids. MALDI MSI was used to monitor the remobilization of amino acids from protein reserve and their localizations over a germination time course in white and brown rice, to identify the timepoint at which the rice germ nutrition value was the highest. In both varieties, GABA localized specifically near the germination area and shifted over time into the coleoptile. GABA was shown to increase constantly and reached a maximum after 96 h. In the white rice variety, most amino acids (e.g., glutamic acid, alanine, arginine, asparagine, aspartic acid, cysteine, glycine, histidine, isoleucine, leucine, lysine, methionine, phenylalanine, proline serine, threonine, tryptophan, tyrosine, and valine) accumulated in the scutellum, the coleoptile, and the aleurone layer but were absent from the endosperm. Except for tryptophan, methionine, and aspartic acid, most of them were found in the endosperm in the brown rice variety [52].

Although considered micronutrients, phospholipids represent up to 10% of rice grain’s lipid content and have a positive effect on human health. As such, it is essential to monitor the content and localization of phospholipids over storage and processing. Twelve phospholipids showing different spatial distribution were identified using MALDI MSI on brown rice grains. Lysophosphatidylcholine (LysoPC) (16:0), LysoPC (18:2), LysoPC (18:1) and LysoPC (18:0) as well as phosphatidylcholine (PC) (16:0/16:1), and PC (16:0/16:0) were found everywhere, especially in the endosperm. In contrast, PC (16:0/18:2), PC (16:0/18:1), PC (18:1/18:3), PC (18:1/18:2) and PC (18:0/18:2) were only located in the bran and in the scutellum. PC (18:0/18:1) was also found at the most external part of the endosperm [53].

MALDI MSI is also competing with more traditional metabolic analysis, allowing comparative studies between different lines as well as nitrogen uptake experiments and nitrogen cycle studies [54,55]. In *Camelina sativa* seeds, a change in the distribution of phospholipid and triacylglycerol could be observed by manipulating the expression of genes involved in lipid metabolisms, such as genes encoding an acyl–acyl carrier protein thioesterase, a fatty acid desaturase, or an elongase [54]. In a radish plant fed with a tea prepared from a fermented radish bulb grown using ^15^N KNO_3_, the integration of ^15^N to new molecules and their becoming could be visualized [55].

The sensitivity of MALDI MSI depends on the ionization of the molecules present in the tissue sections. On maize leaves and roots, derivatization usefulness has proven itself when comparing the genotypes B73 and Mo17 for their composition in primary amines, carbonyl groups, and carboxylic acids metabolites. For example, in combination with gold matrix, 151, 53, and 108 new metabolites were detected using the derivatization reagents coniferyl aldehyde, Girard’s T reagent, and 2-picolylamine, while they were 25, 85, and 70 detected when using DHB as a matrix. In addition, the use of an electrospray deposition system increased the derivatization by Girard’s T reagent by up to 365 new unique features compared to a regular TM Sprayer (Supplementary Table S1) [56]. Besides the high molecular coverage, the multi derivatization workflow was able to precisely localize metabolites in specific compartments of the root and leaves. For example, amino acids such as alanine, valine, asparagine, and glutamine were essentially localized in the root cortex of Mo17, whereas they were also present in the pith of B73, at the center the vascular cylinder. The biological meaning of this specific localization is still unclear, but the authors suggested that it could be related to a different location for amino acid synthesis in the two genotypes. Therefore, amino acids synthetized in the shoot of B73 would be redistributed through the vasculature to the roots, while they would be directly synthetized in the root cortex of Mo17. In the leaf, aminobutyric acid and phosphorylethanolamine were homogeneously distributed throughout the entire tissue, except for the xylem, whereas dotriaconatal was localized in the epidermidis layer as wax lipids. In addition, hexose and hydroxyloxindole acetate glucoside were more homogenously localized, as they were also present in the veins. However, the significance of their specific localizations is still unexplored. Another interesting work used a five-micron, high-resolution MALDI MSI of maize roots to image with high spatial resolutions a disaccharide within the xylem tissues [57].

Plant hormones are essential to regulate plant-microbe interactions and responses to stress, making their detection by MALDI MSI of great interest. Due to the noise generated by the self-ionization of the organic matrix, the detection of small molecules including plant hormones is not always possible. However, this limitation has been overcome by the development of a nanoparticle-based matrix [58]. For example, in rice roots sections, out of nine plant hormones and associated compounds, only five were detected using CHCA as a matrix. The use of iron-based nanoparticles allowed the detection of all nine compounds with a minimum of background noise and lowered the detection limit from 2 to 32 times depending on the plant hormone analyzed. Among the newly detected molecules were auxin, brassinosteroid, and heavy hydrogen-labeled abscisic acid (ABA) (Table 1). On transversal sections made in the elongation area of rice roots, ABA and cytokinins (tZ and iP) were mostly located in the epidermal layers. In contrast, brassinosteroid, salicylic acid, auxin, and the ethylene precursor 1-aminocyclopropane-1-carboxylic acid (ACC) were found at the epidermis, the cortex, and the stele. The localizations of ABA and cytokinins were consistent with a former report on rice root using MALDI MSI and CHCA as a matrix [59]. In rice, Fe_3_O_4_ nanoparticles have also proven to be useful to analyze antimicrobial diterpenoids such as momilactone A and phytocassane B, which are in this case not efficiently ionized by traditional matrices (Table 1) [60]. Those diterpenoids were shown to accumulate specifically in resistant rice plants at the site of infection by *Xanthomonas orizae*, although they were absent in susceptible rice plants.

When looking for internal metabolites, desorption electrospray ionization mass spectrometry is usually used with an expected spatial resolution around 200–300 μm, which is much lower than the 20–30 μm obtained with MALDI MSI. For this reason, the imprinting of samples on porous polytetrafluoroethylene sheets represents a good alternative. MALDI MSI analysis performed on soybean leaves showed that the spatial resolution remained around 30 μm and that the spatial delocalization was limited despite the pressing forces applied during the process [60]. In addition, sample imprinting prevented the drawbacks due to irregularities at the surface of the leaves, which could alter the depth of the acquisition in some local areas. Such a system has proven to be useful to study the molecular processes underlying the soybean—*Aphis glycines* interaction [60] (Supplementary Table S1). During soybean–aphid interaction, insects are known to secrete a honeydew composed of oligosaccharides such as raffinose and stachyose while feeding. The distribution pattern of honeydew deposition around the feeding sites could be visualized with high resolution using MALDI MSI. In addition, nucleobase, amino acids, and phosphocholine were colocalized with dead cells at the feeding point due to the triggering of plant defense mechanisms by aphid saliva effector proteins. In agreement with those observations, the positive regulator of systemic acquired resistance, pipecolic acid, and the insect-induced isoflavones, formononetin and dihydroxyflavones, were shown to accumulate at the feeding site, suggesting a role in local defense responses, while the hormone salicylic acid involved in plant defense priming was observed in both the feeding site and the veins in its vicinity.

**Figure 5:**
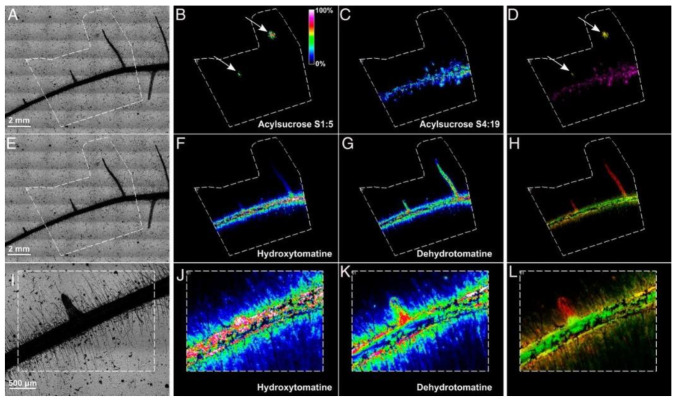
MALDI MSI of the spatial distribution of acyl sugars and steroidal glycoalkaloids exuded in response to rhizosphere microbial communities using positive ion mode. (**A**,**E**,**I**) optical images of the tomato roots analyzed. White dashed lines indicate the region scanned. (**B**,**C**) MALDI MSI of acylsucrose S1:5, (*m/z* 427.18) and acylsucrose S4:19 (*m/z* 665.33), respectively; (**F**,**J**) MALDI MSI of hydroxytomatine (*m/z* 1050.54); (**G**,**K**) MALDI MSI of dehydrotomatin (*m/z* 1032.54); (**D**,**H**,**L**) merge pictures of acyl sugars or steroidal glycoalkaloids. Arrows highlight specific accumulation of metabolites in lateral root tips [62].

When it comes to a plant response to its surrounding environment in soil, metabolites present in the root exudate and released in the rhizospheric space are of great importance. So far, none of the collecting methods are optimal and they barely bring any information in terms of spatial localization. MALDI MSI analysis of root exudate and rhizospheric soil still needs further adaptations, but very promising results have been shown recently that deserve to be highlighted (Supplementary Table S1). For example, by incubating *Arabidopsis* plant on a nylon membrane soaked with medium and using CHCA as a matrix, it was possible to quantify and spatially localize on the root small organic acids such as malate and citrate (Table 1) in response to phosphate deficiency and aluminum toxicity [61]. The transferability of the system was validated on *Marchantia polymorpha* during phosphate deficiency. On the other hand, tomato plants were incubated on a thin layer of agar medium containing 1% of a soil microbial suspension and DHB used as a matrix to study acyl sugars and steroidal glycoalkaloids exudation induced by the rhizosphere microbial communities [62]. This way, it was demonstrated that acylsucrose S1:5 accumulated preferentially on the lateral root tips, whereas acylsucrose S4:19 was predominantly accumulating on the main root hairs (Table 1, Figure 5). Very specific localizations were also observed for steroidal glycoalkaloids (Figure 5). Finally, a polyethylene box with removable side panels was used to cultivate *Panicum virgatum* L. plants in conditions resembling real soil environment and to allow a better accessibility to the root system with a minimum of disturbances [63]. A printout of the rhizosphere interface was generated by applying a wet polyvinylidene fluoride membrane, and the collected metabolites were analyzed using DHB as a matrix in positive ion mode. Among the detected molecules were adenine, carnitine, and arginine (Table 1).

## 5. The Molecular Complexity of Plant–Microbe Interactions

As widely discussed in the previous paragraphs, bacteria can be involved in antagonistic interactions with fungi by producing compounds that can affect fungal growth and development. In contrast, microbial endophytes can colonize the internal part of the plant without causing any harm, but rather bringing benefits to the plant host and sometimes to itself [64]. As such, they sometimes act as biological control agents against plant pathogens. MALDI MSI was used to identify and solve the spatiotemporal production of antimicrobial compounds released by root-colonizing *Bacillus* (e.g., *B. amyloliquefaciens* and *B. subtilis*). Among them, we can cite surfactins, iturins, plipastatin, streptorubin, and fengycin [41,65]. A more extensive list is reported in Supplementary Table S1. More specifically, MALDI MSI coupled with MS/MS analysis was performed on the *B. amyloliquefaciens* antibiome secreted at the level of the biofilm coating the root of *Solanum lycopersicum*. In situ, new surfactins (C14–C15) were discovered and shown to be released at later time points post-inoculation [66] (Table 1).

**Figure 6:**
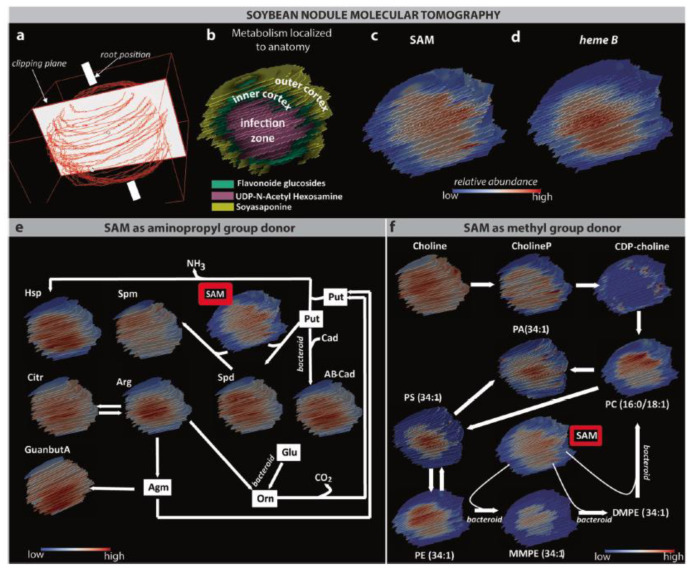
3D MALDI FTICR MSI of soybean root nodule metabolism. (**a**) Building of tomography images from 2D images. (**b**) Microscopic anatomical regions imaged by characteristic compounds: co-localization of UDP-N-acetyl hexosamine with the infection zone, flavonoid glycoside with the inner cortex, and soyasaponin within the outer cortex of the soybean nodule. 3D distribution of (**c**) SAM and (**d**) heme B within the soybean root nodule. 3D distribution within soybean nodules of the (**e**) polyamine and (**f**) PC biosynthesis pathways involving SAM during biological nitrogen fixation [67].

Another great example of plant–bacteria interaction is the symbiosis between soybean root and the nitrogen-fixing soil bacteria *Bradyrhizobium japonicum*. Spatial compartmentalization of metabolites within the nodule was observed via 3D MALDI FTICR MSI applying norharman as a matrix, and the results are shown in Figure 6. Although metabolic homogeneity was until then assumed within the infection zone of the nodule, spatial asymmetry was revealed for the first time for S-adenosyl methionine (SAM) and adenosine diphosphate (ADP). It was hypothesized that SAM and ADP colocalization was maybe correlated with ADP involvement in SAM biosynthesis. As SAM is involved in both PC and polyamine biosynthesis, the spatial localization of downstream metabolites was also investigated. In the polyamine pathway, only spermine showed an asymmetric distribution, whereas in the PC pathway, only PC and phosphatidylethanolamine (PE) were unevenly localized. Moreover, those results illustrated that plant-specific PC biosynthesis through CPD-choline happened essentially in the cortex of the nodules, while bacteroid-specific PC synthesis took place in the infection zone. The change in the spatial distribution and abundance of SAM and ADP when using a rhizobia strain unable to fix nitrogen also supported the idea that those metabolites play a central role in nitrogen fixation [67] (Table 1, Figure 6). A similar example of nitrogen-fixing symbiosis is the one between *Medicago truncatula* and *Sinorhizobium meliloti*. Here, it was possible to perform in situ profiling via MALDI MSI, using a combination of a novel matrix 1,8(dimethyl-amino) naphthalene (DMAN) with the commonly used DHB. A large panel of organic acids, amino acids, sugars, lipids, and flavonoids were identified and are listed in Supplementary Table S1 [68,69]. Among them, asparagine, the exportation form of the nitrogen fixed toward the rest of the plant, was essentially localized on the edge of the nodule, while heme, an essential part of the leghemoglobin insuring optimum low oxygen concentration for nitrogenase activity, localized exclusively at the center of the nodule. Using the same symbiotic system, MALDI Orbitrap MSI was employed to decipher the role of endogenic small peptides during nodule formation and nitrogen fixation. Indeed, the comparison of young seedlings with mature plants showed that some peptides re-localized from the root to the nodules during plant development, suggesting a role in nodule formation and/or function. In addition, three peptides with very different spatial localization inside and around the nodules were absent when using mutant plant developing nonfunctional nodules *dnf1−1*. The first was essentially localized to the root, the second to the root and around the nodule, while the last one was exclusively found inside the nodule. Finally, several ferritins and aquaporins with a potential role in iron incorporation into nitrogenases and water availability management, respectively, were also detected [68,69].

MALDI MSI has also been used to study the response of nitrogen-fixing symbiosis to different environmental stresses such as salt stress and phosphate deficiency. AP MALDI MSI was used to study the metabolites produced in nodules and roots of *M. truncatula* during salt stress conditions. The study revealed an accumulation of arginine inside the nodule and of the triterpenoid soyasaponin I on the outer layer of the nodule, metabolites that have been previously characterized for their role in salt tolerance [70]. Furthermore, spatial differences in the positively charged PC content of nodules from *M. truncatula* were observed under phosphorus limitation using MALDI MSI. For instance, PC (34:2) and PC (36:2) species (Table 1) were detected in lower abundance across the nodule in phosphorus-depletion conditions. PC (38:5), essentially present in the inner part of the nodule in phosphate-sufficient conditions, re-localized toward the proximal area of the nodule under phosphate deficiency. PC (36:4), found in the outer layer of the nodule in presence of phosphate, was located inside the nodule under stress. This suggests that PC species might have a different function depending on their location in the nodule and that nodule membranes undergo lipid remodeling during phosphorus stress to adapt to the new situation [71].

Cyclotides are 27–37 aa residues polypeptides (cyO2, cyO3, cyO13, cyO19, and Kalata S) produced by plants as a part of plant host defense mechanism against fungal pathogens and pests. They present antifungal activity against fungal plant pathogens such as *Fusarium oxysporum* and *B. cinerea*. Using MALDI MSI, cycloviolacin O2, cycloviolacin O3, and cycloviolacin O19 (Table 1) were shown to dominate potential entry points for fungal pathogens, more specifically leaf mesophyll and epidermis, inside and close to the vascular bundle as well as in the pericycle, cortex, and rhizodermis of roots. In contrast, Kalata S (Table 1) was essentially found in petiole parenchyma and leaf mesophyll but not in the root, which was consistent with its presumed activity against pests and lower antifungal activity compared with cyO2, cyO3, and cyO19 [72].

Another great example of antimicrobial molecules produced by the plants to defend themselves against pathogens is phytoalexins. MALDI MSI has been used to characterize the role of several of these metabolites during pathogenic interactions. For instance, on *Vitis vinifera* leaves infected by *Plasmopara viticola*, resveratrol accumulated in a scattered manner on the leaf surface, probably colocalizing with different infection sites. In contrast, Viniferins localized essentially around small veins (Table 1) [73]. In pea pods, the phytoalexin (+)-pisatin and the aromatic compound pinoresinol monoglucoside were detected on the endocarp epidermal cell layer of pea pod infected by *Fusarium solani f.* sp. *phaseoli* (Table 1) [74]. Finally, hesperidin (Table 1) produced by *Citrus limonia* was detected in a much higher concentration in tissues infected by *Xylella fastidiosa* compared with healthy plants, accumulating in xylem, pitch, collenchyma, and epidermis from petioles as well as in xylem, collenchyma, and mesophyll from leaves. MALDI MSI results also helped to support the formation of needle-like crystallized material in infected xylem due to hesperidin as part of a defense mechanism [75].

## 6. Molecular Imaging in Insects, Nematodes, and Worms

**Figure 7:**
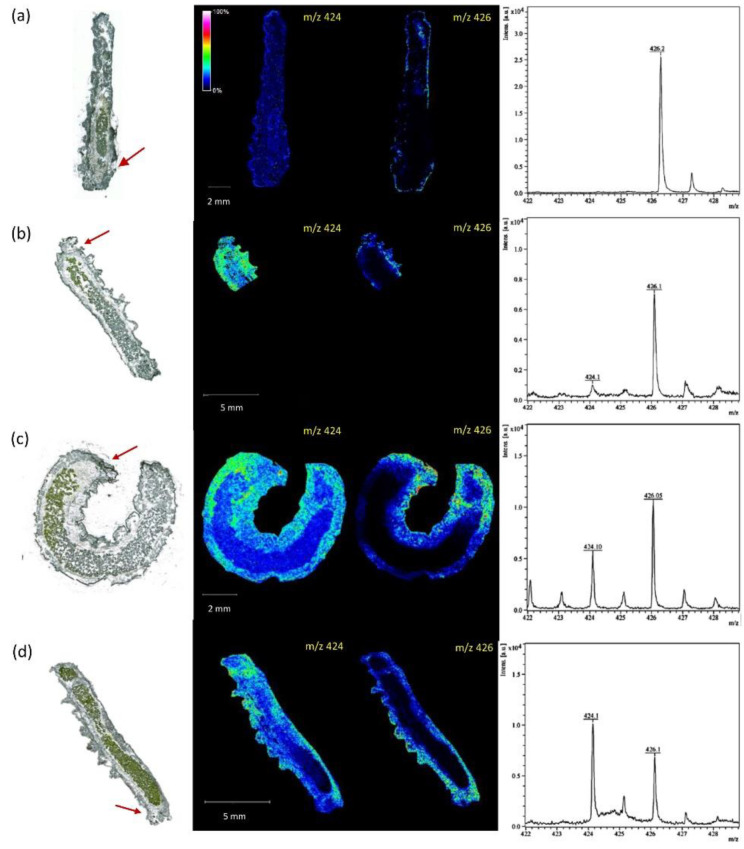
MALDI MSI of deprotonated sinalbin (*m/z* 424) and an unknown metabolite (*m/z* 426) and mass spectrometry analysis of *Athalia rosae* larvae cross-section (15 µm) after ingestion of leaves containing sinalbin (0 min (**a**), 5 min (**b**), 20 min (**c**), and 1 day (**d**), respectively). The head of the larvae is indicated by red arrows [82].

Over the last decade, MALDI MSI has been used to study the biological complexity of several insects, including *Drosophila melanogaster*, *Apis millifera, Cataglyphis nodus, Anopheles stephensi*, and *Periplaneta americana*. The molecular characterizations performed so far have focused essentially on two different molecular classes. Nevertheless, they have provided an extensive and reliable inventory of neuropeptides in brain sections of *A. millifera*, *P. americana*, and *C. nodus* [76,77,78] and lipid compounds in the body sections of *D. melanogaster* and *A. stephensi* [27,79,80] (Supplementary Table S1). In bees, for example, the localization and the concentration of neuropeptides AmTRP-5 and AmAST-1 in the brain depending on their age was associated with their task inside the hive and outdoor [81] (Table 1). In flies, the transfer of the pheromone CH503 from male to female organs during mating could be monitored using MALDI MSI [27] (Table 1). Finally, in *Athalia rosae* larvae, MALDI MSI was used to demonstrate the rapid sequestration of glucosinolate sinalbin in hemolymph upon ingestion, avoiding their activation by plant myrosinases during digestion and further toxicity, as shown in Figure 7 [82] (Table 1). Depending on the organism, sample 3D structure, composition, and sample preservation can be a challenge, affecting the detection and identification of peaks. However, innovations in sample preparation and imaging, as well as computational correction, have proven to be extremely useful to circumvent those drawbacks [83,84,85]. For example, although it was until then considered to be only possible with fresh samples, an adjustment in samples preparation involving partial deparaffinization and the absence of aqueous washing step allowed the characterization of the neuropeptide composition of 30-year-old *P. americana* samples [84].

There are less data related to nematodes and worms. However, a few studies performed on *Caenorhabditis elegans* and *Schistosoma mansoni* showed promising results for lipid detection [83,86,87] (Supplementary Table S1). Moreover, a recent study investigated the effect of the parasitism of tomato roots by the nematode *Meloidogyne incognita* on polypeptides and secondary metabolites, but focused only on the plant side of the interaction [88] (Supplementary Table S1).

Finally, MALDI MSI showed that graphene nanoparticles negatively affect the earthworm *Eisenia fetida* by altering its amino acid, sugar, and polyamine metabolisms. In particular, variations in alanine, phenylalanine, proline, and arginine were associated with potential stress responses, whereas fluctuations in glucose, myo-inositol, and spermidine were associated with a sudden need for energy, a potential destabilization of the osmotic balance, and growth inhibition, respectively [89] (Table 1).

## 7. Animal Tissues as Exemplary for Molecular Imaging: Brain, Testicles, and Kidney

The structure of rat and mouse brain tissue sections, which consist of distinct regions, is an optimal sample to correlate the chemical composition with the specific histological region and, therefore, to investigate the biological environments. In this context, MSI of brain tissue plays a crucial role not only in neurobiology but also in tissue sample preparation development. Many studies evaluated the performance of a novel MALDI MSI method, e.g., evaluation of a new matrix, or instrumental setup by analyzing animal brain tissue and mapping specific histological regions [28,90,91].

Lipid species were shown to have a differential distribution in rat brain tissue sections analyzed by MALDI MSI. The ion intensity obtained by MSI was compared with the ion abundances obtained from LC-MS/MS of the microdissected brain regions. Especially, PC species were identified and localized in several regions of the tissue, showing the different salt content within the histological regions [92].

**Figure 8:**
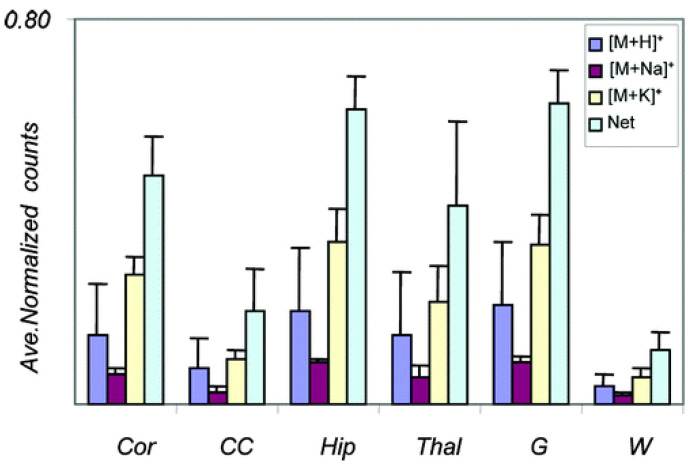
Graphical display of the measured intensity of alkali salt adduct forms of PC (16:0/16:0) ([M + H]^+^, *m/z* 734.6; [M + Na]^+^, *m/z* 756.6; [M + K]^+^, *m/z* 772.6) and their sum averaged over four different MALDI MS images. Cor: cortex, CC: corpus callosum, Hip: hippocampus, Thal: thalamus, G: cerebellum gray, W: cerebellum white [92].

For example, the different forms ([M + H]^+^, [M + Na]^+^, [M + K]^+^) for PC (16:0/16:0) were compared across the brain regions as shown in Figure 8. The adduct [M + K]^+^ is more abundant in the hippocampus than in the corpus callosum. The protonated form instead is more abundant in gray (G) than in the white (W) cerebellum.

Therefore, the salt’s content affects the regional localization of specific lipids. This might reflect a different lipid localization in the cell due to a different exposure to metal alkali under experimental and tissue preparation conditions [92]. Salts content has an important role in biology, especially in neurobiology, in which the gradients of ions modulate neurotransmission. For this reason, the ability to image the salts of lipids in different compartments also allows further investigation in neural physiology. Another example of salts from the PC (36:1) lipid has been reported by using 1,1-binaphthyl-2,2-diamine (BNDM) as a novel matrix, and they showed different ionic maps along the brain regions and lung cancer tissues [93].

The different chemical composition is not only due to the different salts present but also to the composition of the fatty acids involved in phospholipids biosynthesis. As an example, the species PC (16:0/18:1) appeared in the MALDI MS image of rat brain with greater intensity in the gray matter area compared with the white matter. The species PC (18:0/22:6) was distributed exclusively in the cerebellar gray matter. Other PC molecules containing a 22:6 acyl chain (16:0/22:6 [M + H]^+^, *m/z* 806.6; 20:0/22:6 [M + H]^+^, *m/z* 862.6) also had increased abundance in the cerebellum gray matter [92], showing that ionic abundances of the PCs are highly correlated to the histological regions.

It is known that different classes of phospholipids, distinguished by their head group, are localized in different brain regions. For example, the glycerophosphoserine (PS) (22:6/22:6) was revealed by nanostructure initiator MALDI MSI to be abundant in the cerebellum region of the mouse brain [90]. The PS (34:0) was detected in negative mode mainly in the cortex and hypothalamus. Glycerophosphoinositol (PI) (36:4) appeared mainly in the gray matter of the cerebellum, cortex, and hypothalamus. PE (40:6) was localized in the gray matter of the cerebellum, cortex, and hippocampus, and last but not least, sulfatide (ST) (d18:0/C20-OH) was found to be distributed in the white matter of the cerebellum and midbrain sections [95]. In the cerebellum, PI, glycerophosphate (PA), PS, glycerophosphoglycerol (PG), and ST, detected as negative ions, could be used as markers to distinguish the grey from the white matter in brain tissue. More specifically, PA (40:6), PS (36:2), and PI (38:4) were distributed in the gray matter, while sulfatide C18-OH was located in the white matter. Moreover, all the ions shared the same localization at the level of the granular cells layer [95]. Only the species PI (40:6) was found in the granular cell layer but not in the gray matter, and the grey matter regions were differentiated by PI (38:4) and PC (32:0) as observed in other studies [96,97]. This differential phospholipids localization finds an explanation in the lipid processing, especially at the level of the hypothalamus [98].

Another interesting work, which employed a protocol based on ammonium formate washing, is the detection and spatial localization of gangliosides, identified by on-tissue MS/MS, in negative mode on formalin-fixed rat brain tissue. They were mainly localized in the gray matter (cerebral cortex) compared with white matter (corpus callosum) [99].

Other relevant metabolites and neurotransmitters for the central nervous system were localized along the brain tissue regions by high-resolution and high-accuracy MALDI MSI [100]. For example, choline was found to have an ubiquitous distribution explained by its role as an essential nutrient in many animal organs [101]. On the other hand, in other studies, choline was distributed mainly in the intermediate gray layer of the superior colliculus [93]. Acetylcholine (Ach) was imaged higher in the cortex and brainstem but lower in the olfactory bulb and cerebellum [100]. The Ach localization was found to be well correlated with the distribution map of acetylcholinesterases from all brain atlases. Nucleotides such as AMP were ubiquitously distributed in the rat brain with a higher abundance in the cortex [93,100].

**Figure 9:**
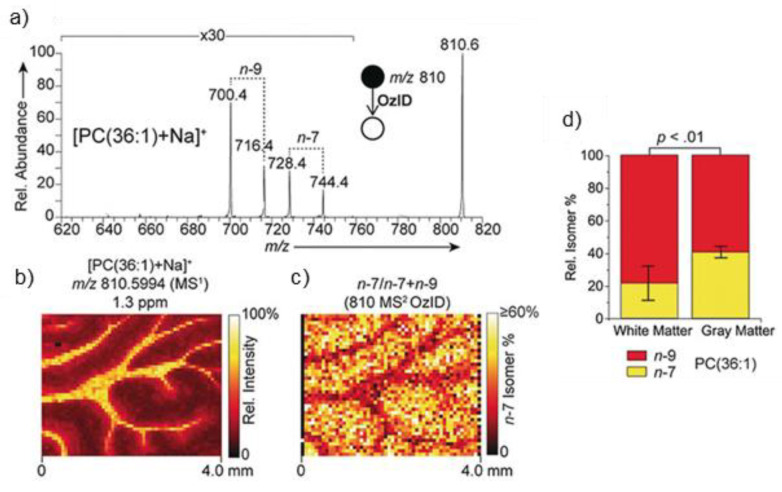
(**a**) MALDI OzID spectrum of [PC (36:1) + Na]^+^ ions revealing the presence of *n*-9 and *n*-7 db isomers, (**b**) The corresponding full-scan FTMS image of the [PC (36:1) + Na]^+^ ion (*m/z* 810.5994) and (**c**) fractional distribution image of *n*-7 and *n*-9 isomers (*n*-7)/(*n*-7 + *n*-9) showing an enrichment of the *n*-7 isomer in the gray matter. (**d**) Graphs show the *n*-7 and *n*-9 relative isomer percentages for PC (36:1). Error bars represent the coefficient of variation from each region (*n* = 5 each for white and gray matter regions) [102].

The most advanced work is the spatial resolving power of lipid isomers by using ozone-induced dissociation in rat brain tissue. Indeed, positional isomers PC (36:1) *n*-7 and PC (36:1) *n*-9 were successfully localized mainly to gray matter and white matter, respectively (Figure 9) [102,103]. Briefly, the collision with ozone leads to the dissociation of the double bond (db) by ozonolysis, producing positional isomer-specific product ions: aldehyde and Criegee ion [104].

Similarly to the brain, testicle tissues have a complex morphology consisting of mainly seminiferous tubules that contain a variety of cell types, such as spermatogonium, spermatocyte, spermatozoon, and Sertoli cells. The biochemistry behind the maturation process of the seminiferous tubules is of great interest because lipids and metabolites are well correlated to the morphological assembly of the tubules. More precisely, the maturation process occurs gradually from the peripheral area to the inner tubules. In this regard, the development-dependent occurrence of seminolipids and their localization were investigated to gain more insight into the germ cell differentiation and reproductive function by MALDI MSI. MALDI MSI has a limitation in the spatial resolution of single cells. Therefore, the ion images were co-registered to stained tissues where the structure of the tubules was classified into layers. Layer 1 includes the basal lamina and is the most peripheral area of the tubules. Layer 2 includes the spermatocyte (middle area of the tubules). Layer 3 includes the inner part of the tubules. Therefore, MALDI-MSI was able to find that seminolipids, which belong to the class of sulfonolipids, have a specific localization in the tubules. In fact, the seminolipids (16:0/16:0), (18:1/16:0), and (18:0/16:0) were localized mainly in the middle and inner layers of the tubules. Single layer-specific seminolipids (14:0/16:0), (16:0/16:1), and (17:0/16:0) were localized in layer 2, 1, and 3, respectively [105,106].

**Figure 10:**
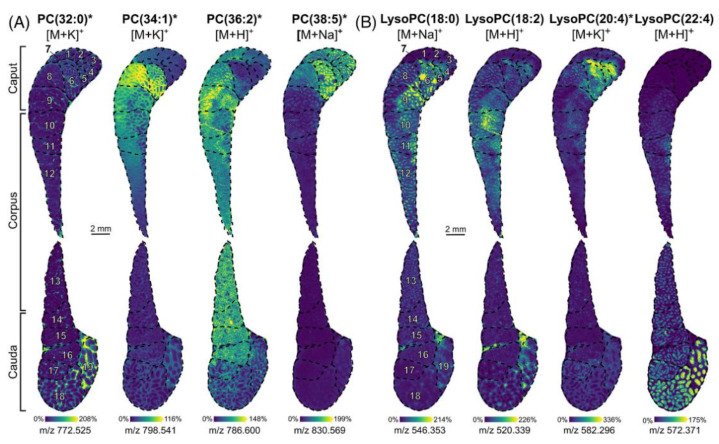
MALDI MSI shows the in situ localization of several PC and LysoPC. Ion images of (**A**) PC (32:0) ([M + K]^+^, *m/z* 772.525), PC (34:1) ([M + K]^+^, *m/z* 798.541), PC (36:2) ([M + H]^+^, *m/z* 786.600), and PC (38:5) ([M + Na]^+^, *m/z* 830.569) and (**B**) LysoPC (18:0) ([M + Na]^+^, *m/z* 546.353), LysoPC (18:2) ([M + H]^+^, *m/z* 520.339), LysoPC (20:4) ([M + K]^+^, *m/z* 582.296), and LysoPC (22:4) ([M + H]^+^, *m/z* 572.371). Ions for which the annotation was confirmed by on-tissue CID are marked with an asterisk. Numbers indicated on the ion images correspond to the 19 intraregional segments. Ion images are presented in veridis color scale automatically adjusted with the hot spot removal option [108].

Recently, high-resolution mass spectrometry imaging in negative and positive mode combined with spatial segmentation and metabolite annotation tools (METASPACE) [107] revealed that lipids and metabolites are involved in the maturation of rat epididymis, which connects the testicle to deferens vas [108]. The morphology of epididymis consists mainly of caput, corpus, and cauda. MALDI MSI could localize in situ several PCs and lysoPCs along different regions (Figure 10).

Lipids belonging to the same structural family have diverse localizations. For example, PC (32:0) was localized in the connective tissues of segments 18 and 19 of the cauda. PC (34:1) was localized in the caput (segments 6, 8, and 9) and corpus (segments 10 to 12). PC (36:2) and PC (38:5) were both moderately abundant in segments 1 to 3 and 8. In detail, PC (36:2) was detected in the smooth muscle and PC (38:5) weakly detected in the sperm stored inside the epididymal tube. LysoPC (18:0) intensity decreased from segments 4 to 15, whereas LysoPC (22:4) intensity increased from segments 9 to 19. LysoPC (18:2) and LysoPC (20:4) were detected in different segments of the caput and corpus but also in the last segments of the cauda. Finally, spatial segmentation also revealed differences in sphingolipids, plasmalogens, and triacylglycerols (TG) composition between caput and cauda.

Testosterone distribution in mouse testes was determined under microscopic level by reactive MALDI MSI in which Girard’s T reagent was employed. The product of the derivatization allowed the authors to trace the distribution of testosterone inside and outside the seminiferous tubules of testis tissue of mice treated with human chorionic gonadotropin [109]. In addition, other androgens such as 5α-dihydrotestosterone (DHT) were identified and spatially localized by using the same derivatization agent. Girard’s T derivatives were identified and localized not only on testes tissues but also on prostate tissue, opening to the MSI procedures the doors to pathological research of cell-specific androgen synthesis in prostate disease [110].

The kidney is an important organ for the excretion of several compounds and has morphological structures that consist of the cortex, the medulla, and the renal pelvis. A MALDI source implemented with a post-ionization laser (MALDI-2) was employed on MSI of mouse kidney tissue to find with high-resolution imaging capabilities lipidic compounds that are histologically specific [111]. Several localizations were found in rat kidneys by tracing three ion channels. [PE (O-36:5) + H]^+^ was localized to the inner medulla, the inner stripe, the glomeruli, and the interstitial regions of the kidney. Both [PE (O-40:8) + H]^+^ and [PC (38:6) + H]^+^ were specific to the kidney tubule, with [PE (O-40:8 + H]^+^ being more abundant in tubules contained within the outer stripe of the medulla, whereas [PC (38:6) + H]^+^ was more abundant in tubules contained within the cortex.

An interesting study regarding the composition of the kidney tissue was performed by using a multifunctional reactive MALDI matrix enabling dual polarity and lipid isomers resolved MS^2^ imaging [112]. The matrix employed was a benzophenone-based compound, 2 benzoylpyridine (BzPy), and it could functionalize the double bonds through the Paterno-Büchi photoreaction. Mouse kidneys were analyzed at high spatial resolution, and lipids were localized and identified along the different histological regions. Dual-polarity high-resolution MALDI MSI found that PS (O-36:1) and PA (36:1) were associated with the inner renal medulla and the renal tubules, while sphingomyelin (SM) (34:1) and glycerophosphoethanolamine ceramide (PE-Cer) (36:1) were localized in the outer renal medullary interstitium regions and the glomeruli. High signal intensities of PE (38:0) and sulfatide hexocyl ceramide (SHexCer) (42:2) were associated with the renal cortex.

**Figure 11:**
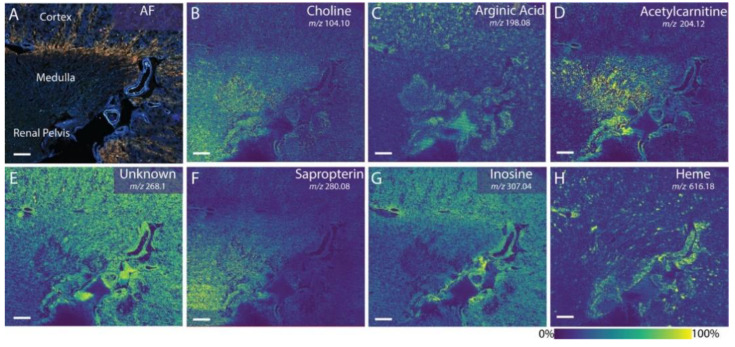
Ion images of small metabolites at 20 μm pixel size correlated to autofluorescence (AF) microscopy (**A**), showing the spatial and size diversity of the detectable analytes (**B**–**H**). Each metabolite localizes to different regions within the kidney, such as the cortex, medulla, and renal pelvis. Scale bars are 1.5 mm [113].

Spatially resolved metabolites and their isomers in human kidneys were reported by the implementation of ion mobility in MALDI MSI experiments [113]. Many metabolites were localized throughout the entire kidney, such as inosine, although several were detected in different segments of the kidney. For example, choline and sapropterin were both detected in high abundance within the medulla and renal pelvis, while acetylcarnitine was most abundant within the medulla and argininic acid was mostly detected within the cortex (Figure 11). Carnitines, involved in fatty acid β-oxidation, were mainly distributed in the renal medulla, whereas glycerophosphocholine was mostly localized in the renal medulla and pelvis [114].

## 8. Conclusions

In the last few years, MALDI MSI has found various applications in different fields of research, e.g., animal and plant biology, entomology, as well as microbiology. In the latter, despite a simpler cell structure, MALDI MSI has been applied to unravel the complex microbial communication network and the correlation between the localization of certain molecules and their biological function.

Due to its high sensitivity and increased throughput, MALDI MSI has been an asset in the biomedical science, to discover new biomarkers for diagnostic purposes as well as to develop new drugs. Indeed, MALDI MSI can provide specific localizations and follow local changes in the distribution of molecules within the tissues of an organism.

Although MALDI MSI applications are still in their infancy in plant research, similar benefits can be observed, as it is helping to better understand the development of plants as well as their association with microorganisms and allowing the development of new treatments against plant pathogens, which could have a relevant impact on modern agriculture.

In conclusion, with this review, we show how MALDI MSI is an excellent tool providing unbiased portraits of the molecular spatial composition of tissues, and how its area of application can only grow in the future as there is a growing need to provide more detailed pictures of complex biological mechanisms.

**Table 1:** List of molecules detected in different organisms using MALDI-MSI and their localizations.

Sample	Compartment	Class of Compound	Matrix	Technique	Reference
Dried agar slices	Microbial extracellular interface	Siderophores	DHB/CHCA (1:1)	MALDI TOF MSI and ion mobility	[26,30,37]
Piece of medium placed on MALDI plate	Interface of fungal colonies	Indol alkaloids	DHB and DHB/CHCA (1:1)	MALDI TOF MSI	[34]
Agar piece placed on MALDI plate	Fungal mycelium	Polyketide	Nanostructured silicon	MALDI TOF MSI on nanosilicone	[25]
Ant thorax section on ITO slide	Ant exoskeleton: propleural plate	Ergothioneine	DHB	MALDI Orbitrap MSI	[32]
Agar section on MALDI plate	Microbial extracellular interface	Quinolones rhamnolipids	DHB/CHCA (1:1)	MALDI FT ICR MSI	[40,41]
Agar section on MALDI plate	Chlamydospores	Cyclo-lipopeptide	DHB/CHCA (1:1)	MALDI MSI	[43]
Cryosection of sclerotia	Sclerotia	Alkaloids	DHB	MALDI MSI (Synapt G2 with ion mobility)	[44]
Dried colonies on the slide	Inhibitory zone at the microbial interface	Solanapyrones, disaccharides, isoflavones, and isoflavonoids	DHB	MALDI FT ICR MSI	[46]
Agar cut on ITO slide	Inhibitory zone	lipopeptaibol	DHB/CHCA (1:1)	MALDI MSI	[48]
Surface tissue section on MALDI plate	Pea pods	Aromatic compound	DHB	MALDI MSI with ion mobility	[74]
Leaf imprints on porous PTFE sheet	Rice root	Diterpenoids	Fe_3_O_4_ nanoparticles	MALDI MSI	[60]
Root on MALDI plate with nylon membrane	Root	Organic acids	CHCA	MALDI MSI Synapt G1	[61]
Lyophilized root on slide	Rhizosphere	Acyl sugars and steroidal glycoalkaloids	DHB	MALDI FT ICR MSI	[62]
PVDF membrane with roots on MALDI plate	Rhizosphere interface	Nucleobase, amino acid, and derivatives	DHB	MALDI FT ICR MSI	[63]
Root-colonized growth on ITO slide	Aerial roots/root biofilm coated	Cyclo-lipopeptides	CHCA	MALDI TOF MSI	[65,66]
Cryosection of root on ITO slide	Root nodule	Choline derivative, organic acid, and amino acids	Norharmane	3D MALDI FTICR MSI	[67]
Cryosection of nodules on plain glass microscope slide	Nodule	Amino acid, triterpenoid, phospholipid, disaccharide, nucleobases, vitamins	DHB and CHCA	AP MALDI MSI Orbitrap	[70,71]
Tissue covered with aluminum foil and embedded in paraffin. Microtome sections on ITO slide	Stem, leaf	flavonoids	DHB/CHCA (1:1)	MALDI TOF MSI	[75]
Cryosection of nodules on glass microscope slide	Nodule	Peptide	DHB and CHCA	MALDI Orbitrap MSI	[69]
Cryosections on ITO slides	Leaf epidermises, vascular bundles, roots, petiole collenchyma, root	Cyclotides	DHB	MALDI TOF MSI	[72]
Leaf fixed with aluminized tape on MALDI plate	Leaf	Stilbenoid and polyphenol	DHB	MALDI TOF MSI	[73]
Cryosections	Brain	Neuropeptide and tryptic peptides	CHCA	MALDI TOF MSI	[76,81]
Cryosections on frosted glass slides	Fly genitals	Pheromones and lipids	DHB	MALDI Orbitrap MSI	[27]
Cryosection on ITO slides	Hemolymph	Glucosinolate	CHCA	MALDI TOF MSI	[82]
Cryosections on ITO slides	Worm	Amino acid, sugars, and polyamine	2-MBT	MALDI TOF MSI	[89]
Cryosections	Rat brain	Phospholipids, neurotransmitters, nucleotides	DHB, nanostructure initiator, DAN, BNDM, and norharmane	MALDI TOF MSI MALDI Orbitrap MSI MALDI Orbitrap with ozonolysis	[92,93,94,95,100,102]
Cryosections	Rat testicles	Seminolipids, phospholipids, and steroids	CHCA, DHB, and AA. Derivatization for steroids.	MALDI TOF MSI MALDI FT ICR MSI iMScope QT	[105,106,108,109,110]
Cryosections	Rat kidney	Phospholipids and small-molecular-weight metabolites	DHB, benzoylpyridine CHCA, and DAN	AP-MALDI Orbitrap MSI MALDI timsTOF MSI MALDI TOF MSI	[112,113,114]

## Data Availability

Not applicable.

## Supplementary Materials

The following are available online at https://www.mdpi.com/article/10.3390/ijms222212393/s1.

## References

[1] Megason S.G., Fraser S.E. (2007). Imaging in Systems Biology. Cell.

[2] Kherlopian A.R., Song T., Duan Q., Neimark M.A., Po M.J., Gohagan J.K., Laine A.F. (2008). A review of imaging techniques for systems biology. BMC Syst. Biol..

[3] Kamweru P.K. (2021). Imaging from anatomic to molecular and atomic resolution scales: A review. Afr. J. Biotechnol..

[4] Spraker J.E., Luu G.T., Sanchez L.M. (2020). Imaging mass spectrometry for natural products discovery: A review of ionization methods. Nat. Prod. Rep..

[5] Dilillo M., Pellegrini D., Ait-Belkacem R., de Graaf E.L., Caleo M., McDonnell L.A. (2017). Mass Spectrometry Imaging, Laser Capture Microdissection, and LC-MS/MS of the Same Tissue Section. J. Proteome Res..

[6] Breuker K., Knochenmuss R., Zenobi R. (1999). Gas-phase basicities of deprotonated matrix-assisted laser desorption/ionization matrix molecules. Int. J. Mass Spectrom..

[7] Knochenmuss R. (2006). Ion formation mechanisms in UV-MALDI. Analyst.

[8] Karas M., Glückmann M., Schäfer J. (2000). Ionization in Matrix-Assisted Laser Desorption/Ionization: Singly Charged Molecular Ions Are the Lucky Survivors. J. Mass Spectrom..

[9] Knochenmuss R. (2016). The Coupled Chemical and Physical Dynamics Model of MALDI. Annu. Rev. Anal. Chem..

[10] McEwen C.N., Inutan E.D., Moreno-Pedraza A., Lu I.C., Hoang K., Pophristic M., Trimpin S. (2021). Sublimation Driven Ionization for Use in Mass Spectrometry: Mechanistic Implications. J. Am. Soc. Mass Spectrom..

[11] Giraldo-Dávila D., Chacón-Patiño M.L., Ramirez-Pradilla J.S., Blanco-Tirado C., Combariza M.Y. (2018). Selective Ionization by Electron-Transfer MALDI-MS of Vanadyl Porphyrins from Crude Oils. Fuel.

[12] Knochenmuss R., Stortelder A., Breuker K., Zenobi R. (2000). Secondary ion-molecule reactions in matrix-assisted laser desorption/ionization. J. Mass Spectrom..

[13] Zhou Q., Fülöp A., Hopf C. (2020). Recent developments of novel matrices and on-tissue chemical derivatization reagents for MALDI-MSI. Anal. Bioanal. Chem..

[14] Corinti D., Crestoni M.E., Fornarini S., Pieper M., Niehaus K., Giampà M. (2019). An integrated approach to study novel properties of a MALDI matrix (4-maleicanhydridoproton sponge) for MS imaging analyses. Anal. Bioanal. Chem..

[15] Horatz K., Giampà M., Qiao Z., Moestue S.A., Lissel F. (2021). Polymerization as a Strategy to Improve Small Organic Matrices for Low-Molecular-Weight Compound Analytics with MALDI MS and MALDI MS Imaging. ACS Appl. Polym. Mater..

[16] Spengler B., Hubert M. (2002). Scanning microprobe matrix-assisted laser desorption ionization (SMALDI) mass spectrometry: Instrumentation for sub-micrometer resolved LDI and MALDI surface analysis. J. Am. Soc. Mass Spectrom..

[17] McDonnell L.A., Heeren R.M.A. (2007). Imaging mass spectrometry. Mass Spectrom. Rev..

[18] Ščupáková K., Balluff B., Tressler C., Adelaja T., Heeren R.M.A., Glunde K., Ertaylan G. (2020). Cellular resolution in clinical MALDI mass spectrometry imaging: The latest advancements and current challenges. Clin. Chem. Lab. Med..

[19] Horatz K., Ditte K., Prenveille T., Zhang K.-N., Jehnichen D., Kiriy A., Voit B., Lissel F. (2019). Cover Feature: Amorphous Conjugated Polymers as Efficient Dual-Mode MALDI Matrices for Low-Molecular-Weight Analytes. ChemPlusChem.

[20] Giampà M., Lissel M.B., Patschkowski T., Fuchser J., Hans V.H., Gembruch O., Bednarz H., Niehaus K. (2016). Maleic Anhydride Proton Sponge as a Novel MALDI Matrix for the Visualization of Small Molecules (<250 m/z) in Brain Tumors by Routine MALDI ToF Imaging Mass Spectrometry. Chem. Commun..

[21] Yuan M., Breitkopf S.B., Yang X., Asara J.M. (2012). A Positive/Negative Ion–Switching, Targeted Mass Spectrometry–Based Metabolomics Platform for Bodily Fluids, Cells, and Fresh and Fixed Tissue. Nat. Protoc..

[22] Buszewska-Forajta M., Patejko M., Macioszek S., Sigorski D., Iżycka-Świeszewska E., Markuszewski M.J. (2019). Paraffin-Embedded Tissue as a Novel Matrix in Metabolomics Study: Optimization of Metabolite Extraction Method. Chromatographia.

[23] Ye H., Gemperline E., Venkateshwaran M., Chen R., Delaux P.-M., Howes-Podoll M., Ané J.-M., Li L. (2013). MALDI mass spectrometry-assisted molecular imaging of metabolites during nitrogen fixation in the *Medicago truncatula–Sinorhizobium meliloti symbiosis*. Plant J..

[24] Dalisay D.S., Kim K.W., Lee C., Yang H., Rübel O., Bowen B.P., Davin L.B., Lewis N.G. (2015). Dirigent Protein-Mediated Lignan and Cyanogenic Glucoside Formation in Flax Seed: Integrated Omics and MALDI Mass Spectrometry Imaging. J. Nat. Prod..

[25] Chen P.Y., Hsieh C.Y., Shih C.J., Lin Y.J., Tsao C.W., Yang Y.L. (2018). Exploration of Fungal Metabolic Interactions Using Imaging Mass Spectrometry on Nanostructured Silicon. J. Nat. Prod..

[26] Vergeiner S., Schafferer L., Haas H., Müller T. (2014). Improved MALDI-TOF microbial mass spectrometry imaging by application of a dispersed solid matrix. J. Am. Soc. Mass Spectrom..

[27] Khalil S.M., Pretzel J., Becker K., Spengler B. (2017). High-resolution AP-SMALDI mass spectrometry imaging of *Drosophila melanogaster*. Int. J. Mass Spectrom..

[28] Horatz K., Giampà M., Karpov Y., Sahre K., Bednarz H., Kiriy A., Voit B., Niehaus K., Hadjichristidis N., Michels D.L. (2018). Conjugated Polymers as a New Class of Dual-Mode Matrices for MALDI Mass Spectrometry and Imaging. J. Am. Chem. Soc..

[29] Feichtmayer J., Deng L., Griebler C. (2017). Antagonistic Microbial Interactions: Contributions and Potential Applications for Controlling Pathogens in the Aquatic Systems. Front. Microbiol..

[30] Boya P.R.A., Martin H.C., Fernandez-Marin H., Gutierrez M. (2019). Fungus-Growing Ant’s Microbial Interaction of *Streptomyces sp*. and *Escovopsis sp*. through Molecular Networking and MALDI Imaging. Nat. Prod. Commun..

[31] Cheah I.K., Halliwell B. (2012). Ergothioneine; antioxidant potential, physiological function and role in disease. Biochim. Biophys. Acta.

[32] Gemperline E., Horn H.A., DeLaney K., Currie C.R., Li L. (2017). Imaging with Mass Spectrometry of Bacteria on the Exoskeleton of Fungus-Growing Ants. ACS Chem. Biol..

[33] Torres M.J., Brandan C.P., Petroselli G., Erra-Balsells R., Audisio M.C. (2016). Antagonistic effects of *Bacillus subtilis* subsp. subtilis and *B. amyloliquefaciens* against *Macrophomina phaseolina*: SEM study of fungal changes and UV-MALDI-TOF MS analysis of their bioactive compounds. Microbiol. Res..

[34] Bai J., Zhang P., Bao G., Gu J.-G., Han L., Zhang L.-W., Xu Y. (2018). Imaging mass spectrometry-guided fast identification of antifungal secondary metabolites from *Penicillium polonicum*. Appl. Microbiol. Biotechnol..

[35] Chung C.L., Huang S.Y., Huang Y.C., Tzean S.S., Ann P.J., Tsai J.N., Yang C.C., Lee H.H., Huang T.W., Huang H.Y. (2015). The Genetic Structure of *Phellinus noxius* and Dissemination Pattern of Brown Root Rot Disease in Taiwan. PLoS ONE.

[36] Raines D.J., Sanderson T.J., Wilde E.J., Duhme-Klair A.K. (2015). Siderophores. Reference Module in Chemistry, Molecular Sciences and Chemical Engineering.

[37] Ho Y.N., Hoo S.Y., Wang B.W., Hsieh C.T., Lin C.C., Sun C.H., Peng C.C., Lin C., Yang Y.L. (2021). Specific inactivation of an antifungal bacterial siderophore by a fungal plant pathogen. ISME J..

[38] Lee Y.J., Perdian D.C., Song Z., Yeung E.S., Nikolau B.J. (2012). Use of mass spectrometry for imaging metabolites in plants. Plant J..

[39] Hoffmann T., Dorrestein P.C. (2015). Homogeneous Matrix Deposition on Dried Agar for MALDI Imaging Mass Spectrometry of Microbial Cultures. J. Am. Soc. Mass Spectrom..

[40] Moree W.J., Phelan V.V., Wu C.-H., Bandeira N., Cornett D.S., Duggan B.M., Dorrestein P.C. (2012). Interkingdom metabolic transformations captured by microbial imaging mass spectrometry. Proc. Natl. Acad. Sci. USA.

[41] Moree W.J., Yang J.Y., Zhao X., Liu W.T., Aparicio M., Atencio L., Ballesteros J., Sánchez J., Gavilán R.G., Gutiérrez M. (2013). Imaging mass spectrometry of a coral microbe interaction with fungi. J. Chem. Ecol..

[42] Soberón-Chávez G., Lépine F., Déziel E. (2005). Production of rhamnolipids by *Pseudomonas aeruginosa*. Appl. Microbiol. Biotechnol..

[43] Spraker J.E., Sanchez L.M., Lowe T.M., Dorrestein P.C., Keller N.P. (2016). *Ralstonia solanacearum* lipopeptide induces chlamydospore development in fungi and facilitates bacterial entry into fungal tissues. ISME J..

[44] Dopstadt J., Vens-Cappell S., Neubauer L., Tudzynski P., Cramer B., Dreisewerd K., Humpf H.-U. (2017). Localization of ergot alkaloids in sclerotia of *Claviceps purpurea* by matrix-assisted laser desorption/ionization mass spectrometry imaging. Anal. Bioanal. Chem..

[45] Kasahara K., Miyamoto T., Fujimoto T., Oguri H., Tokiwano T., Oikawa H., Ebizuka Y., Fujii I. (2010). Solanapyrone synthase, a possible Diels-Alderase and iterative type I polyketide synthase encoded in a biosynthetic gene cluster from *Alternaria solani*. ChemBioChem.

[46] Kim W., Park J.-J., Dugan F.M., Peever T.L., Gang D.R., Vandemark G., Chen W. (2017). Production of the antibiotic secondary metabolite solanapyrone A by the fungal plant pathogen *Ascochyta rabiei* during fruiting body formation in saprobic growth. Environ. Microbiol..

[47] Köhl J., Kolnaar R., Ravensberg W.J. (2019). Mode of Action of Microbial Biological Control Agents Against Plant Diseases: Relevance Beyond Efficacy. Front. Plant Sci..

[48] Liu R., Khan R.A.A., Yue Q., Jiao Y., Yang Y., Li Y., Xie B. (2020). Discovery of a new antifungal lipopeptaibol from *Purpureocillium lilacinum* using MALDI-TOF-IMS. Biochem. Biophys. Res. Commun..

[49] Hu W., Han Y., Sheng Y., Wang Y., Pan Q., Nie H. (2021). Mass spectrometry imaging for direct visualization of components in plants tissues. J. Sep. Sci..

[50] Bednarz H., Roloff N., Niehaus K. (2019). Mass Spectrometry Imaging of the Spatial and Temporal Localization of Alkaloids in Nightshades. J. Agric. Food. Chem..

[51] Deng Y., He M., Feng F., Feng X., Zhang Y., Zhang F. (2021). The distribution and changes of glycoalkaloids in potato tubers under different storage time based on MALDI-TOF mass spectrometry imaging. Talanta.

[52] Kamjijam B., Suwannaporn P., Bednarz H., Jom K.N., Niehaus K. (2021). Elevation of gamma-aminobutyric acid (GABA) and essential amino acids in vacuum impregnation mediated germinated rice traced by MALDI imaging. Food Chem..

[53] Zhang Y.-X., Zhao X.-B., Ha W., Zhang Y.-D., Shi Y.-P. (2021). Spatial distribution analysis of phospholipids in rice by matrix-assisted laser desorption/ionization time-of-flight mass spectrometry imaging. J. Chromatogr. A.

[54] Horn P.J., Silva J.E., Anderson D., Fuchs J., Borisjuk L., Nazarenus T.J., Shulaev V., Cahoon E.B., Chapman K.D. (2013). Imaging heterogeneity of membrane and storage lipids in transgenic *Camelina sativa* seeds with altered fatty acid profiles. Plant J..

[55] Seaman C., Flinders B., Eijkel G., Heeren R.M., Bricklebank N., Clench M.R. (2014). “Afterlife experiment”: Use of MALDI-MS and SIMS imaging for the study of the nitrogen cycle within plants. Anal. Chem..

[56] Dueñas M.E., Larson E.A., Lee Y.J. (2019). Toward Mass Spectrometry Imaging in the Metabolomics Scale: Increasing Metabolic Coverage Through Multiple On-Tissue Chemical Modifications. Front. Plant Sci..

[57] Feenstra A.D., Dueñas M.E., Lee Y.J. (2017). Five Micron High Resolution MALDI Mass Spectrometry Imaging with Simple, Interchangeable, Multi-Resolution Optical System. J. Am. Soc. Mass Spectrom..

[58] Shiono K., Taira S. (2020). Imaging of Multiple Plant Hormones in Roots of Rice (Oryza sativa) Using Nanoparticle-Assisted Laser Desorption/Ionization Mass Spectrometry. J. Agric. Food Chem..

[59] Shiono K., Hashizaki R., Nakanishi T., Sakai T., Yamamoto T., Ogata K., Harada K.I., Ohtani H., Katano H., Taira S. (2017). Multi-imaging of Cytokinin and Abscisic Acid on the Roots of Rice (*Oryza sativa*) Using Matrix-Assisted Laser Desorption/Ionization Mass Spectrometry. J. Agric. Food Chem..

[60] Klein A.T., Yagnik G.B., Hohenstein J.D., Ji Z., Zi J., Reichert M.D., MacIntosh G.C., Yang B., Peters R.J., Vela J. (2015). Investigation of the Chemical Interface in the Soybean-Aphid and Rice-Bacteria Interactions Using MALDI-Mass Spectrometry Imaging. Anal. Chem..

[61] Gomez-Zepeda D., Frausto M., Nájera-González H.-R., Herrera-Estrella L., Ordaz-Ortiz J.-J. (2021). Mass Spectrometry-Based Quantification and Spatial Localization of Small Organic Acid Exudates in Plant Roots under Phosphorus Deficiency and Aluminum Toxicity. Plant J..

[62] Korenblum E., Dong Y., Szymanski J., Panda S., Jozwiak A., Massalha H., Meir S., Rogachev I., Aharoni A. (2020). Rhizosphere Microbiome Mediates Systemic Root Metabolite Exudation by Root-To-Root Signaling. Proc. Natl. Acad. Sci. USA.

[63] Veličković D., Lin V.S., Rivas A., Anderton C.R., Moran J.J. (2020). An approach for broad molecular imaging of the root-soil interface via indirect matrix-assisted laser desorption/ionization mass spectrometry. Soil Biol. Biochem..

[64] Rajendran G., Sing F., Desai A.J., Archana G. (2008). Enhanced growth and nodulation of pigeon pea by co-inoculation of *Bacillus strains* with *Rhizobium spp*. Bioresour. Technol..

[65] Pathak K.V., Keharia H. (2013). Characterization of Fungal Antagonistic Bacilli Isolated from Aerial Roots of Banyan (*Ficus Benghalensis*) Using Intact-Cell MALDI-TOF Mass Spectrometry (ICMS). J. Appl. Microbiol..

[66] Debois D., Jourdan E., Smargiasso N., Thonart P., De Pauw E., Ongena M. (2014). Spatiotemporal monitoring of the antibiome secreted by *Bacillus* biofilms on plant roots using MALDI mass spectrometry imaging. Anal. Chem..

[67] Veličković D., Agtuca B.J., Stopka S.A., Vertes A., Koppenaal D.W., Paša-Tolić L., Stacey G., Anderton C.R. (2018). Observed Metabolic Asymmetry within Soybean Root Nodules Reflects Unexpected Complexity in Rhizobacteria-Legume Metabolite Exchange. ISME J..

[68] Gemperline E., Jayaraman D., Maeda J., Ané J.M., Li L. (2015). Multifaceted investigation of metabolites during nitrogen fixation in *Medicago via* high resolution MALDI-MS imaging and ESI-MS. J. Am. Soc. Mass Spectrom..

[69] Gemperline E., Keller C., Jayaraman D., Maeda J., Sussman M.R., Ané J.M., Li L. (2016). Examination of Endogenous Peptides in Medicago truncatula Using Mass Spectrometry Imaging. J. Proteome Res..

[70] Keller C., Maeda J., Jayaraman D., Chakraborty S., Sussman M.R., Harris J.M., Ané J.-M., Li L. (2018). Comparison of Vacuum MALDI and AP-MALDI Platforms for the Mass Spectrometry Imaging of Metabolites Involved in Salt Stress in *Medicago truncatula*. Front. Plant Sci..

[71] Dokwal D., Romsdahl T.B., Kunz D.A., Alonso A.P., Dickstein R. (2021). Phosphorus deprivation affects composition and spatial distribution of membrane lipids in legume nodules. Plant Physiol..

[72] Slazak B., Kapusta M., Strömstedt A.A., Słomka A., Krychowiak M., Shariatgorji M., Andrén P.E., Bohdanowicz J., Kuta E., Göransson U. (2018). How Does the Sweet Violet (*Viola odorata L*.) Fight Pathogens and Pests—Cyclotides as a Comprehensive Plant Host Defense System. Front. Plant Sci..

[73] Becker L., Carré V., Poutaraud A., Merdinoglu D., Chaimbault P. (2014). MALDI mass spectrometry imaging for the simultaneous location of resveratrol, pterostilbene and viniferins on grapevine leaves. Molecules.

[74] Seneviratne H.K., Dalisay D.S., Kim K.W., Moinuddin S.G., Yang H., Hartshorn C.M., Davin L.B., Lewis N.G. (2015). Non-host disease resistance response in pea (*Pisum sativum*) pods: Biochemical function of DRR206 and phytoalexin pathway localization. Phytochemistry.

[75] Soares M.S., da Silva D.F., Forim M.R., da Silva M.F., Fernandes J.B., Vieira P.C., Silva D.B., Lopes N.P., de Carvalho S.A., de Souza A.A. (2015). Quantification and localization of hesperidin and rutin in *Citrus sinensis* grafted on *C. limonia* after *Xylella fastidiosa* infection by HPLC-UV and MALDI imaging mass spectrometry. Phytochemistry.

[76] Catae A.F., da Silva Menegasso A.R., Pratavieira M., Palma M.S., Malaspina O., Roat T.C. (2019). MALDI-imaging analyses of honeybee brains exposed to a neonicotinoid insecticide. Pest Manag. Sci..

[77] Ly A., Ragionieri L., Liessem S., Becker M., Deininger S.-O., Neupert S., Predel R. (2019). Enhanced Coverage of Insect Neuropeptides in Tissue Sections by an Optimized Mass-Spectrometry-Imaging Protocol. Anal. Chem..

[78] Habenstein J., Schmitt F., Liessem S., Ly A., Trede D., Wegener C., Predel R., Rössler W., Neupert S. (2021). Transcriptomic, peptidomic, and mass spectrometry imaging analysis of the brain in the ant *Cataglyphis* nodus. J. Neurochem..

[79] Khalil S.M., Römpp A., Pretzel J., Becker K., Spengler B. (2015). Phospholipid Topography of Whole-Body Sections of the *Anopheles stephensi* Mosquito, Characterized by High-Resolution Atmospheric-Pressure Scanning Microprobe Matrix-Assisted Laser Desorption/Ionization Mass Spectrometry Imaging. Anal. Chem..

[80] Yang E., Gamberi C., Chaurand P. (2019). Mapping the fly Malpighian tubule lipidome by imaging mass spectrometry. J. Mass Spectrom..

[81] Pratavieira M., da Silva Menegasso A.R., Garcia A.M.C., dos Santos D.S., Gomes P.C., Malaspina O., Palma M.S. (2014). MALDI Imaging Analysis of Neuropeptides in the Africanized Honeybee (*Apis mellifera*) Brain: Effect of Ontogeny. J. Proteome Res..

[82] Abdalsamee M.K., Giampà M., Niehaus K., Müller C. (2014). Rapid incorporation of glucosinolates as a strategy used by a herbivore to prevent activation by myrosinases. Insect Biochem. Mol. Biol..

[83] Kompauer M., Heiles S., Spengler B. (2017). Autofocusing MALDI mass spectrometry imaging of tissue sections and 3D chemical topography of nonflat surfaces. Nat. Methods.

[84] Paine M.R.L., Ellis S.R., Maloney D., Heeren R.M.A., Verhaert P. (2018). Digestion-Free Analysis of Peptides from 30-Year-Old Formalin-Fixed, Paraffin-Embedded Tissue by Mass Spectrometry Imaging. Anal. Chem..

[85] Kaftan F., Vrkoslav V., Kynast P., Kulkarni P., Böcker S., Cvačka J., Knaden M., Svatoš A. (2014). Mass spectrometry imaging of surface lipids on intact *Drosophila melanogaster* flies. J. Mass Spectrom..

[86] Robert F.M., Chaevien S.C., Louis A.S., Arthur S.E., Richard A.Y. (2015). MALDI Mass Spectrometric Imaging of the Nematode *Caenorhabditis elegans*. Curr. Metabolomics.

[87] Hameed S., Ikegami K., Sugiyama E., Matsushita S., Kimura Y., Hayasaka T., Sugiura Y., Masaki N., Waki M., Ohta I. (2015). Direct Profiling of the Phospholipid Composition of Adult *Caenorhabditis Elegans* Using Whole-Body Imaging Mass Spectrometry. Anal. Bioanal. Chem..

[88] Barbosa E.A., Bonfim M.F., Bloch C., Engler G., Rocha T., de Almeida Engler J. (2018). Imaging Mass Spectrometry of Endogenous Polypeptides and Secondary Metabolites from Galls Induced by Root-Knot Nematodes in Tomato Roots. Mol. Plant Microbe Interact..

[89] Zhang Y., Qin L., Sun J., Chen L., Jia L., Zhao J., Yang H., Xue K., Wang X., Sang W. (2020). Metabolite Changes Associated with Earthworms (*Eisenia Fetida*) Graphene Exposure Revealed by Matrix-Assisted Laser Desorption/Ionization Mass Spectrometry Imaging. Ecotoxicol. Environ. Saf..

[90] Li B., Sun R., Gordon A., Ge J., Zhang Y., Li P., Yang H. (2019). 3-Aminophthalhydrazide (Luminol) as a Novel Matrix for Dual-Polarity MALDI MS Imaging. Anal. Chem..

[91] Belov M.E., Ellis S.R., Dilillo M., Paine M.R.L., Danielson W.F., Anderson G.A., de Graaf E.L., Eijkel G.B., Heeren R.M.A., McDonnell L.A. (2017). Design and Performance of a Novel Interface for Combined Matrix-Assisted Laser Desorption Ionization at Elevated Pressure and Electrospray Ionization with Orbitrap Mass Spectrometry. Anal. Chem..

[92] Hankin J.A., Murphy R.C. (2010). Relationship between MALDI IMS Intensity and Measured Quantity of Selected Phospholipids in Rat Brain Sections. Anal. Chem..

[93] Sun C., Liu W., Mu Y., Wang X. (2020). 1,1′-binaphthyl-2,2′-diamine as a novel MALDI matrix to enhance the in situ imaging of metabolic heterogeneity in lung cancer. Talanta.

[94] Ivanisevic J., Epstein A.A., Kurczy M.E., Benton P.H., Uritboonthai W., Fox H.S., Boska M.D., Gendelman H.E., Siuzdak G. (2014). Brain Region Mapping Using Global Metabolomics. Chem. Biol..

[95] Angel P.M., Spraggins J.M., Baldwin H.S., Caprioli R. (2012). Enhanced Sensitivity for High Spatial Resolution Lipid Analysis by Negative Ion Mode Matrix Assisted Laser Desorption Ionization Imaging Mass Spectrometry. Anal. Chem..

[96] Wang H.-Y.J., Post S.N.J.J., Woods A.S. (2008). A minimalist approach to MALDI imaging of glycerophospholipids and sphingolipids in rat brain sections. Int. J. Mass Spectrom..

[97] Murphy R.C., Hankin J.A., Barkley R.M. (2009). Imaging of lipid species by MALDI mass spectrometry. J. Lipid Res..

[98] Bruce K.D., Zsombok A., Eckel R.H. (2017). Lipid Processing in the Brain: A Key Regulator of Systemic Metabolism. Front. Endocrinol..

[99] Harris A., Roseborough A., Mor R., Yeung K.K.C., Whitehead S.N. (2020). Ganglioside Detection from Formalin-Fixed Human Brain Tissue Utilizing MALDI Imaging Mass Spectrometry. J. Am. Soc. Mass Spectrom..

[100] Ye H., Wang J., Greer T., Strupat K., Li L. (2013). Visualizing Neurotransmitters and Metabolites in the Central Nervous System by High Resolution and High Accuracy Mass Spectrometric Imaging. ACS Chem. Neurosci..

[101] Zeisel S.H., da Costa K.-A. (2009). Choline: An essential nutrient for public health. Nutr. Rev..

[102] Paine M.R.L., Poad B.L.J., Eijkel G.B., Marshall D.L., Blanksby S.J., Heeren R.M.A., Ellis S.R. (2018). Mass Spectrometry Imaging with Isomeric Resolution Enabled by Ozone-Induced Dissociation. Angew. Chem. Int. Ed..

[103] Claes B.S.R., Bowman A.P., Poad B.L.J., Young R.S.E., Heeren R.M.A., Blanksby S.J., Ellis S.R. (2021). Mass Spectrometry Imaging of Lipids with Isomer Resolution Using High-Pressure Ozone-Induced Dissociation. Anal. Chem..

[104] Brown S.H.J., Mitchell T.W., Blanksby S.J. (2011). Analysis of Unsaturated Lipids by Ozone-Induced Dissociation. Biochim. Biophys. Acta.

[105] Goto-Inoue N., Hayasaka T., Zaima N., Setou M. (2012). Imaging mass spectrometry reveals changes of metabolites distribution in mouse testis during testicular maturation. Surf. Interface Anal..

[106] Goto-Inoue N., Hayasaka T., Zaima N., Setou M. (2009). The specific localization of seminolipid molecular species on mouse testis during testicular maturation revealed by imaging mass spectrometry. Glycobiology.

[107] Palmer A., Phapale P., Chernyavsky I., Lavigne R., Fay D., Tarasov A., Kovalev V., Fuchser J., Nikolenko S., Pineau C. (2017). FDR-Controlled Metabolite Annotation for High-Resolution Imaging Mass Spectrometry. Nat. Methods.

[108] Lagarrigue M., Lavigne R., Guével B., Palmer A., Rondel K., Guillot L., Kobarg J.H., Trede D., Pineau C. (2020). Spatial segmentation and metabolite annotation involved in sperm maturation in the rat epididymis by MALDI imaging mass spectrometry. J. Mass Spectrom..

[109] Shimma S., Kumada H.-O., Taniguchi H., Konno A., Yao I., Furuta K., Matsuda T., Ito S. (2016). Microscopic visualization of testosterone in mouse testis by use of imaging mass spectrometry. Anal. Bioanal. Chem..

[110] Cobice D.F., Livingstone D.E.W., Mackay C.L., Goodwin R.J.A., Smith L.B., Walker B.R., Andrew R. (2016). Spatial Localization and Quantitation of Androgens in Mouse Testis by Mass Spectrometry Imaging. Anal. Chem..

[111] Bowman A.P., Bogie J.F.J., Hendriks J.J.A., Haidar M., Belov M., Heeren R.M.A., Ellis S.R. (2020). Evaluation of Lipid Coverage and High Spatial Resolution MALDI-Imaging Capabilities of Oversampling Combined with Laser Post-Ionisation. Anal. Bioanal. Chem..

[112] Wäldchen F., Mohr F., Wagner A.H., Heiles S. (2020). Multifunctional Reactive MALDI Matrix Enabling High-Lateral Resolution Dual Polarity MS Imaging and Lipid C=C Position-Resolved MS^2^ Imaging. Anal. Chem..

[113] Neumann E.K., Migas L.G., Allen J.L., Caprioli R.M., Van de Plas R., Spraggins J.M. (2020). Spatial Metabolomics of the Human Kidney using MALDI Trapped Ion Mobility Imaging Mass Spectrometry. Anal. Chem..

[114] Sun C., Liu W., Geng Y., Wang X. (2020). On-Tissue Derivatization Strategy for Mass Spectrometry Imaging of Carboxyl-Containing Metabolites in Biological Tissues. Anal. Chem..

